# Comparative study of hyperpure chlorine dioxide with two other irrigants regarding the viability of periodontal ligament stem cells

**DOI:** 10.1007/s00784-020-03618-5

**Published:** 2020-10-12

**Authors:** Orsolya Láng, Krisztina S. Nagy, Julia Láng, Katalin Perczel-Kovách, Anna Herczegh, Zsolt Lohinai, Gábor Varga, László Kőhidai

**Affiliations:** 1 Department of Genetics, Cell- and Immunobiology, Semmelweis University, Budapest, Hungary; 2 Department of Oral Biology, Semmelweis University, Budapest, Hungary; 3 Laboratory of Nanochemistry, Department of Biophysics and Radiation Biology, Semmelweis University, Budapest, Hungary; 4 Department in Community Dentistry, Semmelweis University, Budapest, Hungary; 5 Department of Conservative Dentistry, Semmelweis University, Budapest, Hungary

**Keywords:** Chlorine dioxide, Dental stem cells, PDLSC, Viability, Toxicity, Hydrogen peroxide, Chlorhexidine

## Abstract

**Objectives:**

Periodontal ligament stem cells (PDLSCs) have an underlined significance as their high proliferative capacity and multipotent differentiation provide an important therapeutic potential. The integrity of these cells is frequently disturbed by the routinely used irrigative compounds applied as periodontal or endodontic disinfectants (e.g., hydrogen peroxide (H_2_O_2_) and chlorhexidine (CHX)). Our objectives were (i) to monitor the cytotoxic effect of a novel dental irrigative compound, chlorine dioxide (ClO_2_), compared to two traditional agents (H_2_O_2_, CHX) on PDLSCs and (ii) to test whether the aging factor of PDLSC cultures determines cellular responsiveness to the chemicals tested.

**Methods:**

Impedimetry (concentration-response study), WST-1 assays (WST = water soluble tetrazolium salt), and morphology analysis were performed to measure changes in cell viability induced by the 3 disinfectants; immunocytochemistry of stem cell markers (STRO-1, CD90, and CD105) measured the induced mesenchymal characteristics.

**Results:**

Cell viability experiments demonstrated that the application of ClO_2_ does not lead to a significant decrease in viability of PLDSCs in concentrations used to kill microbes. On the contrary, traditional irrigants, H_2_O_2_, and CHX are highly toxic on PDLSCs. Aging of PLDSC cultures (passages 3 vs. 7) has characteristic effects on their responsiveness to these agents as the increased expression of mesenchymal stem cell markers turns to decreased.

**Conclusions and clinical relevance:**

While the active ingredients of mouthwash (H_2_O_2_, CHX) applied in endodontic or periodontitis management have a serious toxic effect on PDLSCs, the novel hyperpure ClO_2_ is less toxic providing an environment favoring dental structure regenerations during disinfectant interventions.

**Electronic supplementary material:**

The online version of this article (10.1007/s00784-020-03618-5) contains supplementary material, which is available to authorized users.

## Introduction

For the last two decades, the discovery of the dental stem cells (DSCs) has opened new perspectives in regenerative dentistry and medicine. The first source of these oral cells with mesenchymal stem cell (MSC) properties was the human dental pulp [1], and subsequently, four more types of DSCs were gained from different tooth-related tissues, such as pulp of exfoliated deciduous teeth [2], periodontal ligament (PDL) [3], dental follicle [4], and apical papilla [5]. The common feature of these DSCs is that they exhibit fibroblast-like morphology with good proliferative potential and fulfill the minimal criteria of MSC characteristics, such as adherence to plastic surface, expression of certain surface antigens (e.g., more than 95% of the cell population express CD73, CD90, and CD105, and less than 2% express hematopoietic markers), and capacity for multipotent differentiation in vitro [6]. Recent studies provided evidence for a wide range of plasticity of these SCs and their ability to repair tooth-related tissues or bone in vivo. Additionally, as DSCs are easily accessible and lack strict ethical concern conversely from their embryonic counterparts, they represent favorable tools also for the therapy of neurodegenerative disorders (e.g., Alzheimer or Parkinson diseases) or cardiac ischemia [7, 8].

Of the abovementioned DSCs, the PDLSCs are of great significance both in theoretical and practical aspects. Due to the lack of consensus criteria defining dental stem cells based on the surface antigen expression pattern, in most of the studies, PDLSCs are characterized by positivity for MSC markers. PDLSCs were found to express the STRO-1 antigen [3, 9]—identified first in bone marrow stromal cells [10]—and other MSC markers, such as CD13, CD29, CD44, CD59, CD90, and CD105 [7]. However, some investigation also revealed embryonic stem cell marker positivity (e.g., Oct-4) of these cells [9]. PDLSCs exhibit multipotent differentiation capacity. In vitro, these cells are able to differentiate into osteogenic, adipogenic [3], chondrogenic [11], neurogenic [12], and myogenic [13] lineages. In vivo, they have fundamental importance in the physiology of PDL, which does not only anchor the cementum covering the root to the alveolar bone but also contributes to its nutrition, homeostasis, and repair. The significant regenerative potential of PLDSCs allows these cells to contribute the spontaneous or medically induced restorative mechanisms of the periodontal region [14, 15]. The differentiation potential of these stem cells is similar to pericytes, while their immunomodulatory character is also well described [16, 17]. The integrity of these cells in the PDL is vital for the whole life of the tooth. One of the longstanding goals of dental care is to keep the periodontium in good health and to reconstruct it when destroyed by the periodontal disease. Therefore, it is of paramount importance to know the effect of the disinfectant substances used routinely in the oral cavity on the physiological processes of these stem cells.

In conventional dental care, irrigative agents are frequently used to eliminate the bacteria from different regions, e.g., outer root surface in case of periodontal or root canal surface in endodontic treatment. Due to the multispecies composition of the biofilm, the effective antibacterial irrigative have a relatively broad spectrum and multiple intracellular targets, which reduce the frequency of resistant cases. Topical antiseptics, such as chlorhexidine (CHX) and hydrogen peroxide (H_2_O_2_), which are routinely applied as disinfectants in dentistry, correspond to these criteria. However, antiseptics, unlike antibiotics, are potentially toxic not only to the infectious microbes but to the host cells as well. Recently, a well-known biocide, chlorine dioxide (ClO_2_), has been invented in the dental care, as its application was suggested for disinfection of the air of dental offices [18] and also of dental instruments [19].

H_2_O_2_ has been acknowledged as an effective disinfecting tool in the dentistry for centuries. Nowadays, it is used in 3% for hemostasis and maximum in 6% in EU or 38% in the USA for bleaching [20]. But the concentration- and time-dependent cytotoxic effects of H_2_O_2_ are well described in human PDL cells [20]. RANK ligand-induced activation was described as potential trigger mechanism of this action besides its free radical activity [21].

CHX is a cationic diphenyl compound that acts as a broad-spectrum bactericidal agent. It is effective against both Gram-positive and Gram-negative bacteria and fungi as well. Based on its chemical characteristics, CHX interacts with the anionic phosphate residue of the lipid molecules and causes severe damage to the cytoplasmic membrane and the peptidoglycan layer of microorganisms [22]. Its therapeutic concentration used in clinical dentistry is in the range 0.05–0.2% as an oral rinse and in 0.2–6% as endodontic irrigation. Even low concentrations of CHX (0.0001%) has been shown to be toxic for gingival fibroblasts, reducing the production of collagen and non-collagen proteins [23]. In human PDL cells, the cytotoxic effects of CHX are concentration- and time-dependent and associated with protein synthesis inhibition [24]. Other studies show that the survival rate of equine fibroblasts increases linearly with decreasing concentrations of CHX, with 50% of survival at 0.005% CHX [25]. Exposure of dental root surfaces to CHX (0.12%) significantly inhibited subsequent attachment of gingival fibroblasts [26]. In addition, the attachment of PDL cells onto the root surface and their morphology were adversely altered with 0.2 to 2.0% CHX pretreatment of the root surface [27].

Thus, we would need a disinfectant which is less toxic for humans but is still effective against the microbes. Lubbers and coworkers [28] have shown in 1982 that chlorine dioxide, “the ideal biocide” [29], might meet these criteria. The use of chlorine dioxide as an antiseptic was hindered, because ClO_2_ solutions of that time were contaminated with other chemicals and were not stable enough. In 2006, however, a new membrane separation process was invented [30], which can produce hyperpure and therefore significantly more stable ClO_2_ solutions [31]. Such solutions are commercially available in Hungary since 2008 under the name of Solumium (https://www.solumium.com/) and applied in dental care since that time [32]. Their favorable antimicrobial efficacy was demonstrated against oral pathogen bacteria [33, 34]. ClO_2_ can be also used as intracanal bleaching substance [24]. It was also demonstrated to be less toxic to equine fibroblasts than CHX [21]. The cytotoxic effect of ClO_2_ on human gingival fibroblasts was shown to be only in the millimolar range (LD_50_ = 0.16 mM), while low concentrations of ClO_2_ did not induce apoptotic responses [25]. ClO_2_ is a size-selective antimicrobial agent [35] which explains theoretically why ClO_2_ solutions lethal for microbes are not harmful to humans. Thus, the remarkable selectivity of ClO_2_ between humans and bacteria is based not on their different biochemistry, but on their different sizes. Based on its previously discussed properties, hyperpure chlorine dioxide solutions should be highly suitable for medical and dental applications. However, a major obstacle of the widespread human clinical application is that practically no published data are available about the possible toxic effects of ClO_2_ on dental cells.

Thus, the purposes of the present study were (i) to compare the cell physiological effects (cytotoxicity and viability) of the three dental irrigative compounds (H_2_O_2_, CHX, and ClO_2_) in human PDLSCs and (ii) to monitor how the aging of PDLSC cultures affect the cellular response to the irrigative chemicals tested.

## Materials and methods

### Antiseptic agents

Analytically pure, aqueous solutions of H_2_O_2_ (3%) and CHX (0.2%) were obtained from the Central Apotheke of the Semmelweis University. The applied concentrations of these antiseptic agents were selected according to their doses applied in the routine clinical practice in periodontology. High-purity ClO_2_ (Solumium™, Solumium Ltd., Hungary) (0.025%) was prepared by a novel membrane technology [35] at the Department of Physics, Budapest University of Technology and Economics. For periodontal treatments and oral rinse, 1:10 dilution of the abovementioned ClO_2_ solution is suggested in human applications.

### Cell isolation and cultures

Periodontal ligament stem cells (PDLSCs) were isolated from impacted, healthy third molars of healthy young adults. Tooth extractions were carried out according to the guidelines approved by the Ethical Committee of the Hungarian Medical Research Council at the Department of Oral Diagnostics, Semmelweis University. We have obtained written consent from each patient. This study was approved by the Semmelweis University Regional and Institutional Committee of Science and Research Ethics. The numbers of the ethical permissions are as follows: 17458/2012/EKU, 25459-4/2019/EKU – ETT-TUKEB. Stem cells were isolated as we described previously [36], with some modifications. In brief, the periodontal ligament was removed by a sterile scalpel and digested in collagenase type I (Sigma Ltd., St. Louis) dissolved in Dulbecco’s phosphate buffer saline (DPBS, Lonza) solution (1 mg/mL) for 1 h at 37 °C. After that, the remaining tissue pieces were pushed through a 22-G needle to loosen the tissue structure and gain single cell cultures. Stem cell cultures were maintained under standard conditions in the alpha modification of Eagle’s medium (αMEM, Gibco) supplemented with 10% fetal bovine serum (FBS, Gibco), 2 mM L-glutamine (Sigma Ltd., St.Louis, USA), 100 units/mL penicillin, and 100 mg/mL streptomycin (Gibco). Subconfluent cultures were passaged weekly at a ratio 1:20 with 0.05% trypsin-EDTA (Gibco). For the experiments, cell populations from the P3 and P7 passages were used. Characterization of PDL stem cells isolated in this way was already described [36], too. The most important characteristics of these cells were (i) increased proliferative capacity, (ii) expressing STRO-1 mesenchymal stem cell marker, (iii) osteogenic differentiation, and (iv) transient deurodifferentiation.

### Concentration-response study

To evaluate the concentration-dependent effects of the 3 antiseptic agents (H_2_O_2_, CHX, and ClO_2_), 9 different dilutions of each agent were prepared with growth medium (from 3.91 × 10^−6^ to 10^−3^ M, by 1:2 dilution in each step). As ClO_2_ is photosensitive and volatile, its dilution series were made in dark Eppendorf tubes which were opened only for the inevitably required time. After a 48-h-long incubation, cell viability was assessed by WST-1 test and impedimetric measurement, respectively (see below). The average inhibitory concentration (IC_50_) values corresponding to the 50% viable cells or 0.5 normalized CI were determined from the concentration-effect curves in the case of the two methods, respectively.

### Cell viability assays

#### Impedimetry

The effect of irrigative agents on the viability of the PDLSC cells was assessed using the xCELLigence SP System (Roche Applied Science, Indianapolis, IN, USA), which monitors the cellular events by measuring electrical impedance across interdigitating gold microelectrodes integrated on the bottom of tissue culture plates (E-plates). The detected impedance is influenced by the viability and morphology of the attached cells on the surface of the electrodes. The presence of cells due to their insulating plasma membranes constrain the electrical current and lead to an increase in the electrode impedance. The impedance depends on the number of the attached cells and on the dimensional change of the attached cells on the electrodes. More cells attached to the electrode or spreading cause a larger increase in the impedance. The change in impedances represented as “cell index” (CI) which is a relative and dimensionless value. Briefly, the experimental protocol was as follows: to register the background value, 100 μL of culture medium was added to each well and impedance was recorded for 1 h to gain constant background curves of impedance. In the following step, PDLSCs were seeded in 10^5^ cells/mL density. After 24-h incubation, they were treated with the solutions of the investigated irrigative compounds (H_2_O_2_, CHX, and ClO_2_) for 10 min and the medium served as reference. The viability of the PDLSC was monitored for 96 h. Normalized impedance to the control calculated by the integrated software (RTCA 1.2—Roche Applied Science, Indianapolis, IN, USA) was used for the statistical evaluation. Each data point represents the mathematical average of three parallels.

#### WST-1 assay

This assay is dedicated to measuring the viability of PDLSCs [37]. In 96-well culture plates, 10,000 PDLSCs were seeded. After 24 h, samples were treated with the irrigative compounds, and final concentrations were H_2_O_2_ 0.3%, CHX 0.02%, and ClO_2_ 0.0025% and 0.00025% solutions in 4-4 parallel wells. After 10 min, all the irrigative agents were removed and the samples were washed three times with normal growth medium, simulating the diluting effect of saliva in the oral cavity during the periodontal treatments. Forty-eight hours after the treatments, the cell viability was measured by the cell proliferation reagent containing water-soluble tetrazolium salt, WST-1 [2-(4-Iodophenyl)-3-(4-nitrophenyl)-5-(2,4-disulfophenyl)-2H-tetrazolium] (Roche). This assay determines mitochondrial dehydrogenase enzyme activity resulting water-soluble cleavage product. The WST-1 reagent was used at 1:20 dilution and incubation time was 2 h at 37 °C. Absorbance was measured at 450 nm with a reference wavelength of 655 nm by a microplate reader (BIO-RAD Model 3550). Background control values were determined in wells seeded with no cells. Each value represents the average of minimum 3 parallel measurements.

#### Morphological studies

Cellular morphology was observed under an inverted phase-contrast microscope (Nikon TMS). Photomicrographs were taken applying a high-performance CCD camera (COHU) and the Scion image software.

#### Immunocytochemistry

PDLSC cultures grown in 8 chamber slides (Nunc Lab-Tek) were treated with the antiseptic agents similar to the viability studies. After 48 h, treated and untreated cell cultures were fixed in 4% PFA in PBS for 20 min at room temperature (RT). To block non-specific binding, 5% goat serum in PBS was applied for 1 h at RT. Then cell cultures were incubated with the primary antibodies diluted 1:100 with 2.5% goat serum in PBS overnight at 4 °C. The applied primary antibodies were the following: anti-STRO-1 antibody (type mouse IgM)—a generous gift from Prof. Richard Oreffo (University of Southampton, UK); anti-CD90 (Thy-1; type mouse IgG) and the anti-CD105 (endoglin; type mouse IgM) antibodies purchased from Calbiochem and Santa Cruz Biotechnology, respectively. Goat anti-mouse IgM and IgG type secondary antibodies conjugated with Alexa Fluor 488 (Molecular Probes) were diluted with 2.5% goat serum in PBS in 1:2000 and 1:1000 respectively. The incubation time was 60 min at RT. Finally, the walls of the chambers were removed and the slides were mounted with ProLong Gold antifade reagent with DAPI (Molecular Probes). Immunocytochemically labeled cell cultures were investigated under a fluorescence microscope (Nikon Eclipse E600), the photos were taken by a Retiga 2000R digital CCD camera (QImaging), and analyzed by the Image Pro software (Media Cybernetics). Free macros for automated fluorescence analysis of image processing software FIJI/ImageJ were applied to evaluate immunostaining of stem cell markers. Each test point represents 3 × 3 reading of the samples.

### Statistical evaluation of data

Data are expressed as the arithmetical means ± standard errors of the mean (SEM) from minimum 3 independent experiments with 3-6 parallels. Statistical evaluation of the data was performed by the STATISTICA 10 software applying Kruskal-Wallis test (the non-parametric analog of the ANOVA). A difference was considered statistically significant if *P* < 0.05.

## Results

### Time-course effects

**Fig. 1:**
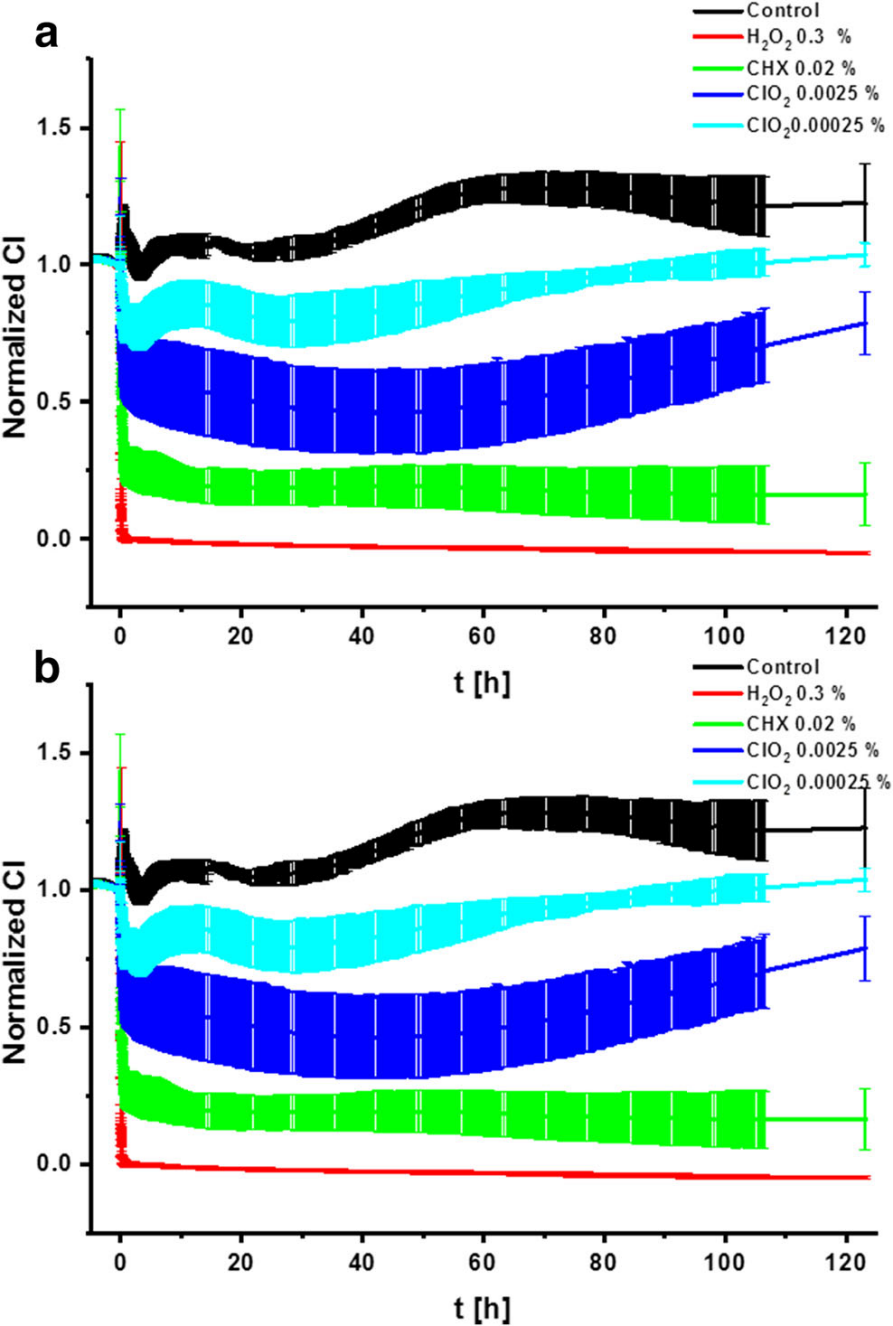
The curves of the normalized cell index (CI) value represent the short- and long-term effects of the irrigative agents on PDLSCs recorded by impedimetric analysis. Short-term (2 h) (**a**) and long-term (120 h) (**b**) toxic effects of H_2_O_2_ (red), CHX (green), and ClO_2_ (blue and light blue) on PDLSCs applied at 0.1× concentrations used in clinical routine (0.3%, 0.02%, and 0.0025% or 0.00025%)

The impedimetric analysis is a dedicated technique to register small differences in cell density in a real-time system. Due to the high sensitivity of the assay, we could evaluate short- and long-term effects elicited by the three antiseptic agents (H_2_O_2_, CHX, and ClO_2_) (Fig. 1). In short-term (0–2 h) detection (Fig. 1a), a rapid decrease of normalized CI profile was recorded in 0.3% H_2_O_2_ and 0.02% CHX treatments which effects were identical to strong surface membrane level cytotoxic effects resulting loss in electrical insulating capacity. High and low concentrations (0.0025% and 0.00025%) of ClO_2_ worked differently; however, a decrease of normalized CI was still observed, but this compound was significantly less toxic compared to the other two antiseptic substances. In long-term relations (2–120 h) (Fig. 1b), treatments with 0.3% H_2_O_2_ and 0.02% CHX retained their short-term cytotoxic effects, while the characteristics of curves in samples treated with 0.00025% and 0.0025% ClO_2_ differed as a consistent increase of the curves (0.00025%—30 h; 0.0025%—50 h) was detected, which points out the presence of a surviving and proliferating subpopulation of cells.

### Concentration-response study

**Fig. 2:**
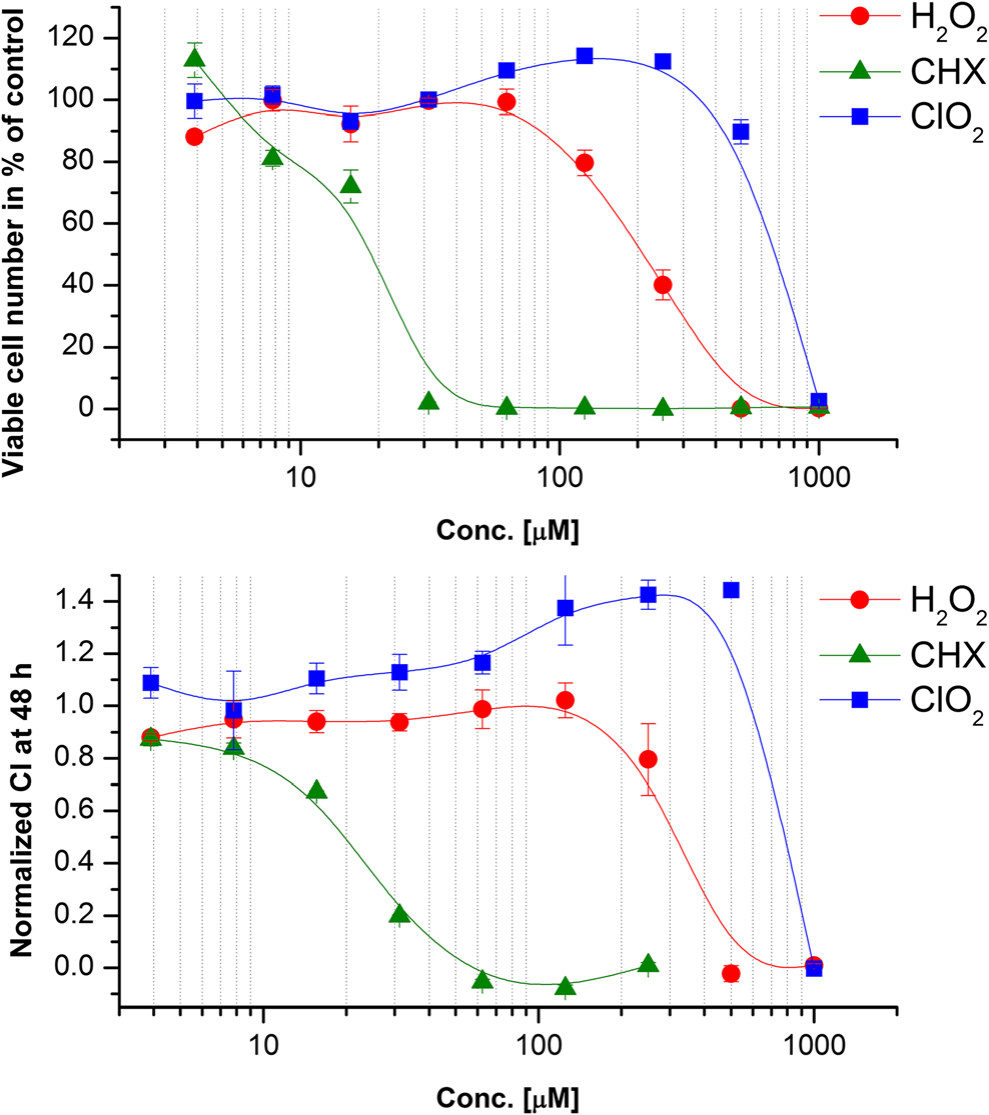
Dose-response curves of PDLSCs treated with irrigative agents: H_2_O_2_, CHX, and ClO_2_. Analysis of 48-h cultures by **a** WST-1 test and **b** impedimetric analysis

The concentration dependence of the cytotoxic effects was evaluated in two independent assays: (i) WST-1 detects viability of cells on the basis of mitochondrial-dehydrogenase activity and (ii) impedimetrics evaluate the number of living cells on the basis of electrical insulator ability of the surface membrane. The concentration range study showed that the two assays which are based on independent cellular mechanisms led to remarkably overlapping results (Fig. 2a, b). Ranking of the threshold concentration sensitivity of PDLSCs to of irrigative compounds showed the following order of sensitivity: CHX >> H_2_O_2_ > ClO_2_. The calculated IC_50_ values related to the highest concentration applied in periodontology are presented in Table 1. The IC_50_ were well comparable in the two different assays applied: (i) WST-1 test: IC_50_ (CHX) = 29.5 μM < IC_50_ (H_2_O_2_) = 209 μM < IC_50_ (ClO_2_) = 638 μM; (ii) impedimetry: IC_50_ (CHX) = 36.4 μM < IC_50_ (H_2_O_2_) = 327 μM < IC_50_ (ClO_2_) = 795 μM. Calculating proportions from the appropriate pair of concentrations (clinically applied concentration/IC_50_) shows that ClO_2_—compared to H_2_O_2_ and CHX—has the most advantageous ratio. The concentration applied in dental praxis is a 4.7- to 5.8-fold of the IC_50_ determined by different assays in the present experiment.

**Table 1:** The calculated IC_50_ values of the irrigative compounds tested and their clinically applied concentrations in periodontology

Compound	In vitro		Clinical		Clinical/IC_50_ WST-1 test	Clinical/IC_50_ impedimetry
	IC_50_ WST-1 test (μM)	IC_50_ impedimetry (μM)	Applied concentration in periodontology			
			%	μM		
H_2_O_2_	209	327	3.5	88,240	422	269
CHX	16.6	20.5	0.25	3960	238.5	193
ClO_2_	638	795	0.025	3700	5.8	4.65

The efficacy of the compounds can be estimated by the ratio of clinically applied, curative concentration/IC_50_

### Morphology/viability studies

**Fig. 3:**
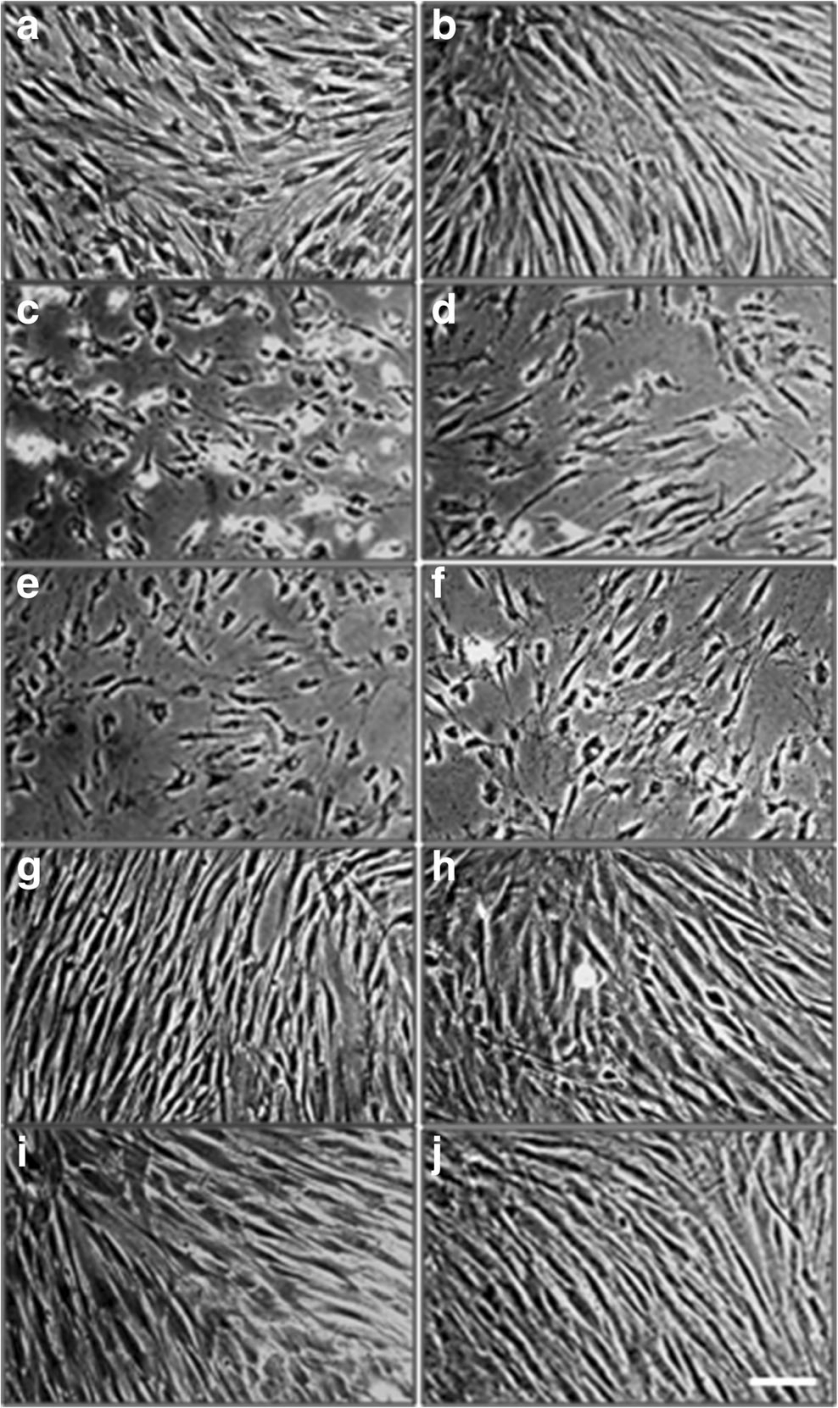
Phase contrast microscopical study of PDLSC cultures from passage 3 (**a**, **c**, **e**, **g**, **i**) and passage 7 (**b**, **d**, **f**, **h**, **j**). Changes in morphology compared to the untreated control (**a**, **b**) were evaluated 48 h after the treatment with 0.3 % H_2_O_2_ (**c**, **d**), 0.02 % CHX (**e**, **f**), 0.0025 % ClO_2_ (**g**, **h**), and 0.00025 % ClO_2_ (**i**, **j**). All photomicrographs were taken at the same magnification. Bar indicates 100 μm

In cellular targets during dental treatments, the changing sensitivity of cells might be a major limiting factor. The PDLSC cultures were investigated under a phase-contrast microscope 48 h after treatments with 0.3% H_2_O_2_, 0.02% CHX, 0.0025% ClO_2_, or 0.00025% ClO_2_. Our morphological observations are in line with the abovementioned data. The untreated cell cultures (Fig. 3a) demonstrate healthy fibroblast-like morphology. The deteriorating effects of CHX and H_2_O_2_ were well detectable (Fig. 3b, c). The number of cells dramatically decreased in response to these treatments and the originally spindle-shaped cells become more rounded and their processes get much thinner. These observations collectively indicate a drastic decline in cell viability. In contrast, ClO_2_ did not provoke morphological changes compared to the control group (Fig. 3d, e).

**Fig. 4:**
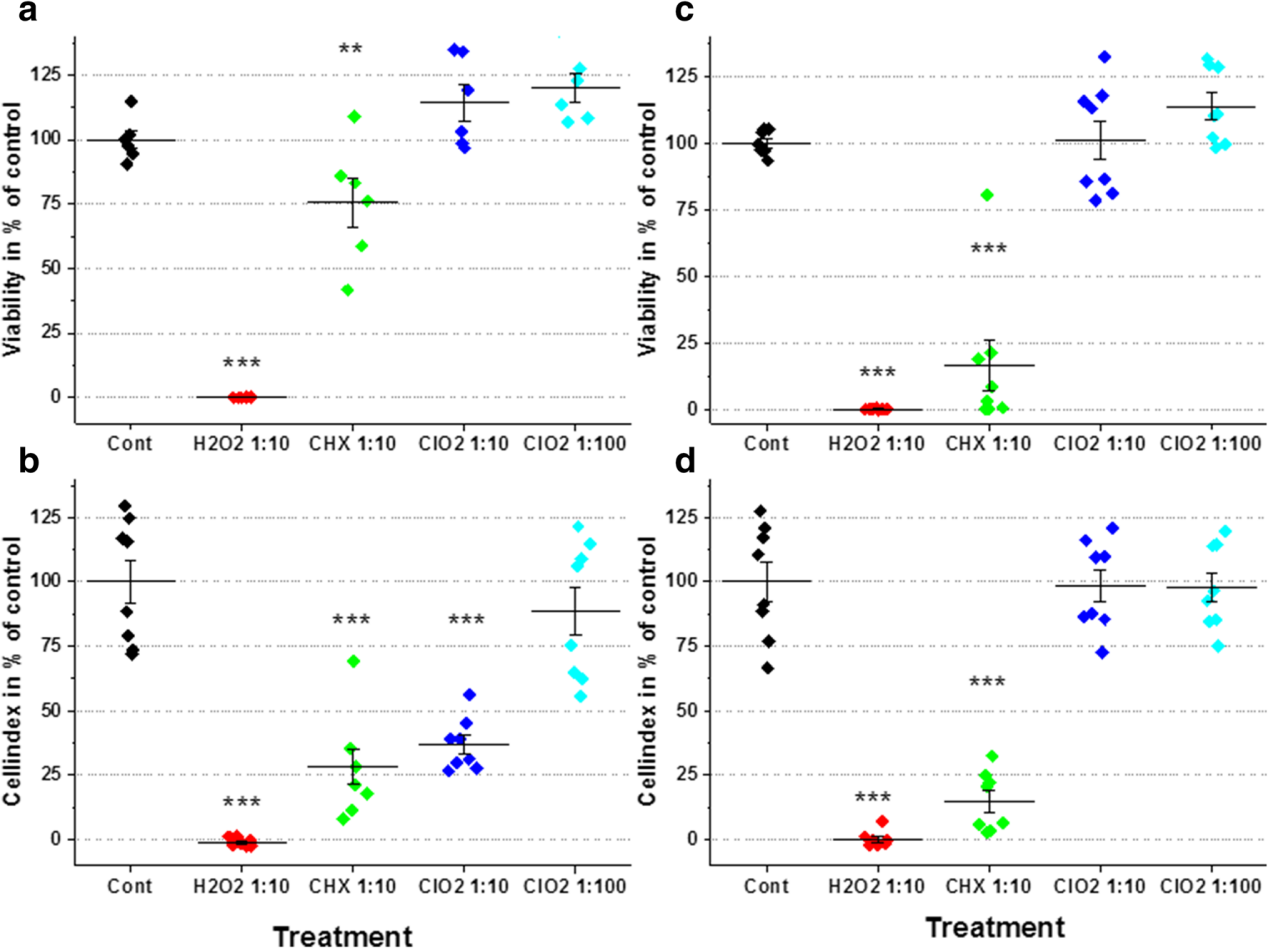
The viability of the PDLSC cultures from passage 3 (**a**, **b**) and passage 7 (**c**, **d**) after 48-h treatment with irrigative agents (H_2_O_2_ 1:10 = 0.3 % H_2_O_2_; CHX 1:10 = 0.02 % CHX; ClO_2_ 1:10 = 0.0025 % ClO_2_ and ClO_2_ 1:100 = 0.00025 % ClO_2_). The cell numbers were assayed by WST-1 test (**a**, **c**) and by impedimetry (**b**, **d**); the values (± SD) are expressed in % of the untreated controls. (**p < 0.01; ***p < 0.001)

In WST-1 assay and impedimetry, H_2_O_2_ elicited the strongest cytotoxic effect, resulting in a complete lack of any viable cells in both P3 and P7 samples. Application of CHX also resulted in significantly deteriorating effects on the viability of PDLSC cultures of both generations (Fig. 4). The lower concentration of ClO_2_ was unequivocally neutral in P3 and P7 cells in both WST-1 and impedimetry assays. The higher applied concentration of ClO_2_ seemed to have no effect only in the WST-1 test, but it showed a significant inhibitory effect in the P3 PDLSC samples by impedimetry (Fig. 4b).

In stem cell marker tests, the expression of three mesenchymal stem cell markers (STRO-1, CD90, and CD105) were assayed as a reference to find out whether the application of the irrigative compounds has any influence on the stem cell characteristics of PDLSCs in different passage levels (P3 and P7).

**Fig. 5:**
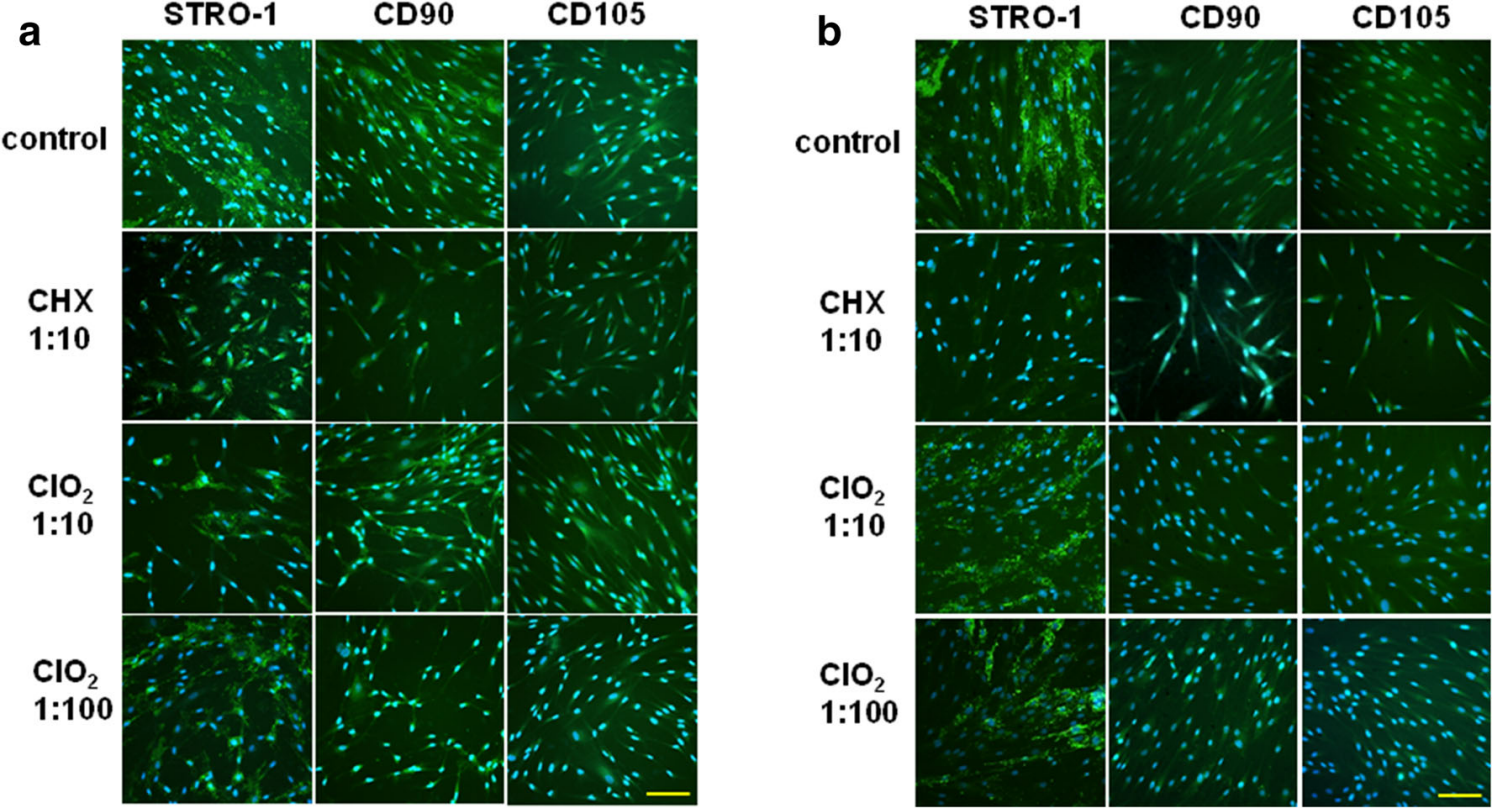
Immunofluorescent detection of the mesenchymal stem cell markers STRO-1, CD90, and CD105 in control (**a**) and PDLSC (**b**) cultures 48 h after the treatment with irrigative agents CHX (0.02%) and ClO_2_ (0.0025 or 0.00025%). (The cultures treated with H_2_O_2_ could not be fixed for immunocytochemistry as all the cells were dead and detached from the slide.) All the photomicrographs were taken at the same magnification—bar indicates 100 μm

Our indirect immunocytochemistry-based evaluations show that none of the tested chemicals provoked characteristic changes in the expression of the aforementioned stem cell markers either in P3 or in P7 generation of the PDLSC cultures. Around 50% of the cells are STRO-1-positive and near 100% of the cells express the CD90 and CD105 markers both in control and treated groups (Fig. 5). Quantitative evaluations of immunocytochemistry (Table 2) in the treated PDLSC cultures show that the CHX treatment resulted in an increased expression of all the 3 stem cell markers (CD105 > CD90 >> STRO-1) in P3 cells while in P7 cells, the CHX treatment resulted a significant decrease in the expression of all the 3 stem cell markers compared to the expression on P3 cells. In P3 cells, the applied ClO_2_ concentrations (1/1 and 1/10) caused a significant increase in the markers STRO-1 and CD105, while in CD90, this effect was observed only at the lower (1/10) concentration. In contrast, in P7 cells, a significant decrease of the markers was observed compared to P3 cells (except CD90 where ClO_2_ 1/1 resulted in a control level expression of the marker).

**Table 2:** Effect of treatment with 0.02% chlorhexidine (CHX), 0.0025% (1/1), or 0.00025% (1/10) ClO_2_ on the expression of stem cell markers of PDL stem cells in passage 3 (P3) and passage 7 (P7)

Stem cell marker	Treatment	P3		P7	
		Fluo. int. [%] Control = 100%	± SD	Fluo. int. [%] Control = 100%	± SD
STRO-1	CHX	130.52*	6.55	86.02	4.15
	ClO_2_ (1/1)	216.52***	10.12	120.43*	3.32
	ClO_2_ (1/10)	145.78**	5.55	114.69	5.41
CD90	CHX	199.23***	7.32	30.98***	3.71
	ClO_2_ (1/1)	83.07	4.44	97.74	4.02
	ClO_2_ (1/10)	159.23**	5.11	54.92***	4.31
CD105	CHX	218.18***	9.71	104.81	6.09
	ClO_2_ (1/1)	213.90***	12.10	55.46***	3.92
	ClO_2_ (1/10)	127.80*	3.22	15.74***	2.76

*p < 0.05; **p < 0.01; ***p < 0.001

## Discussion

There is a wide variety of irrigative agents applied as active ingredients of mouthwashes (e.g., NaF, CPC, xylitol, volatile oil extracts). Nevertheless, H_2_O_2_ and CHX are considered to be the most frequently used components due to their strong antiseptic, analgesic, and anti-inflammatory effect in the oral cavity.

The strong oxidant H_2_O_2_ is applied in dentistry because of its advantageous whitening, disinfectant, and hemostatic properties. Its mechanism of action is based on the H_2_O_2_ penetration into the target cell where its generated oxygen radicals destroy the cell lipids and proteins of the membranes as well as the DNA in the nucleus. Also, in low concentrations, H_2_O_2_ has apoptotic effects. It was considered as a wide range antimicrobial substance; however, its effective concentration range is much higher than that of CHX as its effectiveness is reduced in Gram-positive bacteria by catalase activity [38]. Therefore, their in vitro sporicide effects are evident only in really high (10–30%) concentrations when the incubation time is long [38]. According to the recent regulations of the European Union, only low concentrations of H_2_O_2_ are approved for human use as its wound-healing inhibitory and scarring effects were detected. For tooth whitening, at present, the maximal approved dose is 6% in Europe [39].

CHX is applied as salts of chlorohexidine, and its most frequently used derivative is chlorohexidine-di-glyconate. The positively charged CHX molecules have high affinity to the negatively charged molecular targets such as surface membrane and bacterial cell wall, thereby disturbing their integrity and resulting in bacterial cell lysis [22]. An additional advantageous property of CHX is that it adheres to both hard and soft oral tissues, which results in prolonged effects. The instant and sustained bactericide and bacteriostatic effects of CHX inhibit the development of biofilm and also help to remove the already persisting one. The adverse side effect of CHX is that the precipitation of proteins of the ruined bacteria provoke discoloration of teeth [22].

ClO_2_ has been applied in dental offices for many years for sterilization of the air [18] or dental instruments [19]. However, the direct use of ClO_2_ for human oral treatments was limited since the purity level of the available preparations had been rather low. Additionally, this volatile molecule could not be preserved for long term as the concentration of reactive oxygen radicals drops relatively fast after application [35]. Moreover, the conventional manufacturing process of ClO_2_ is accompanied by the formation of toxic by-products. All of these problems have been solved by a novel membrane filtration technology, which resulted in a product, Solumium™, containing an only highly pure and stable form of ClO_2_ that also has longer stability compared to previously produced unpurified compounds [35]. As a result, the effective local concentration of ClO_2_ can be sustained over a long period of time.

A major message of the present work is the high concentration difference in which ClO_2_ exerts its toxic effects on human PLD-derived cells and microbes. The exact reason(s) for this is (are) not known but the parallel existence of several mechanisms can be regarded.

(i) Free radicals reach the molecular or cellular targets by size-limited diffusion [35].
(ii) The radicals targeting to proteins of membranes and cytoplasm are acting selectively on cysteine, methionine, tryptophan, and tyrosine residues [40].
(iii) Reactions with Fe^2+^ and Mn^2+^ ions or glutathione also limit its effect [40–43].
(iv) The cytotoxic effects of ClO_2_ may appear only in the size range of microbes, but not in eukaryote cells. In microbes, the low amount of cell surface proteins limits the neutralization of ClO_2_ molecules, while the significantly bigger eukaryotic cells are only superficially damaged [35].
(v) Furthermore, in some fields of application where tissues cannot be directly reached, for example, in dentistry, it is also essential that ClO_2_ can transform to gaseous state so that it can easily penetrate into complex structures such as the biofilm or the dentin in periodontal treatment or root canal disinfection, respectively [33, 34].
(vi) In contrast to antibiotics, a special advantage of the ClO_2_ application is that it acts through mechanisms for which the microorganisms cannot develop resistance in a classical sense [35].

In the present study, we compared the cytotoxic effects of the hyperpure ClO_2_, manufactured by new semipermeable membrane technology, with the effects of H_2_O_2_ and CHX, which are routinely applied in periodontal treatments and mouthwashes. The influence of the abovementioned three antiseptics on PDLSC cultures was evaluated by several cell biological methods. According to our impedimetric results, H_2_O_2_ has the strongest and fastest cytotoxic effect as it killed all the cells within 1 min. CHX caused moderate and somewhat slower cell death killing 50% of the cells in 5 min and about 70% during the first half an hour. On the contrary, ClO_2_ exhibited the slightest cytotoxic effect killing only 20–30% of the cells (depending on the concentration applied) during the first 30 min of direct exposure (Fig. 1a). The long-term impedimetric analyses revealed that after ClO_2_ treatment, the proliferation of the remaining cell population can compensate cell loss, indicating that the negative action of this antiseptic agent was temporary. Nevertheless, after CHX treatment, no sign of repopulation was observed during the 5-day-long period, referring to that these cell cultures lost their regeneration potential permanently (Fig. 1b).

In agreement with the impedimetric analysis, our morphological study also proved that H_2_O_2_ and CHX have stronger cytotoxic effects on PDLSC cultures than ClO_2_. We observed on the microphotographs that H_2_O_2_ and CHX treatments led to large-scale cell death whereas the cell could retain their healthy, fibroblast-like morphology after ClO_2_ treatment. The WST-1 viability assay confirmed the previous results showing complete and moderate loss of viable cells after H_2_O_2_ and CHX treatments, respectively, but no change in viability 2 days after ClO_2_ treatment. By qualitative and quantitative evaluation of immunohistochemistry demonstrated that application of CHX and ClO_2_ results in increased expression of the three stem cell markers (STRO-1, CD90, and CD105), nevertheless, the concentration dependence of the process is more fine-tuned in ClO_2_, while effect of CHX seems to be coarsely controlled.

Many papers reported the strong cytotoxic effects of CHX and H_2_O_2_ (e.g., low IC_50_ values) in the concentration ranges of clinical applications even on the non-target, healthy cells of the patient [20, 21, 23–25, 44]. Our present data obtained by WST-1 test and impedimetry provide evidence for these harmful effects even on PDLSC (Fig. 2). However, the novel hyperpure preparation ClO_2_ has only a very weak toxic effect on oral/dental cells (oral epithelium), in spite of its antimicrobial effects reported previously [35]. Studying the direct antibacterial and the biofilm-dissolving effects of CHX and ClO_2_, the group of one of us found that approximately 10 times lower concentration of ClO_2_ is needed to evoke the same or even stronger effect than that of CHX [33, 34]. Similarly, the concentration suggested for oral disinfectant applications is 0.02% for ClO_2_ and 0.2% for CHX [45]. Very importantly, on PDLSCs, the cytotoxic potency difference between CHX and ClO_2_ turns to the reverse and overshoots: the viability assay yielded an ~ 21 times higher IC_50_ concentration for ClO_2_ compared to CHX. Thus, ClO_2_ is 10 times more potent killing bacteria than CHX but 21 times less potent to kill human PDL cells providing an approximately 200 times gap between the two compounds when their selectivity is regarded. This fact should give a great advantage to ClO_2_ over CHX and other traditionally used disinfectant compounds in human oral applications.

Genotoxicity of traditional disinfectants used in dentistry is well known from several prior studies [46–48]. Some experiments performed on the ClO_2_ have also proved potential genotoxicity on buccal epithel; however, these studies evaluated the ClO_2_ only as a component of a complex mouth rinse where additive and synergistic effects of more active ingredients can be assumed rightly. In these studies, the alcohol consumption habits of the patients were not recorded even [29].

These characteristics of the ClO_2_ seem to be advantageous for dental application. Additionally, with respect to the clinical outcome, it is important to preserve dental stem cells during the interventions in order to achieve normal healing. Thus, ClO_2_ could be applied for various purposes such as an effective disinfectant acting on different components (e.g., *Streptococcus mutans*, *Lactobacillus acidophilus*, *Enterococcus faecalis*, or *Candida albicans*) of dental biofilm [34, 49] and acting on endodontium as the irrigative substance of root canal [45]. In the case of root canal biofilms containing mostly anaerobic bacteria, the antibacterial effectiveness of ClO_2_ was similar or lower compared to other, widely used irrigating substances like CHX or NaOCl [50] but here the ClO_2_ loss due to its volatility was supposedly not taken into account. According to Tanner [51], however, ClO_2_ is more effective against planktonic bacteria than other disinfectants (except ozone). Moreover, ClO_2_ is a very selective antibacterial agent (reacts only with few amino acids) and can penetrate lipid membranes more easily than other antimicrobials like HOCl for example [35]. As a consequence, chlorine dioxide should be more effective against biofilms than any other disinfectants.

The aging of the PDLSC cultures (passage numbers) and sensitivity to the tested substances showed a substance-specific correlation in both WST-1 test and impedimetry analysis. The passage number–independent and absolute toxic effect of H_2_O_2_ was manifested in the disintegration of structural elements, presumably induced by the activation of the corresponding key role signaling pathway of RANK ligand and by the direct free radical activity of H_2_O_2_ [23]. The sensitivity to CHX showed a more marked and passage number–related correlation which was reflected in the measured intracellular mitochondrial enzyme activities. In the case of ClO_2_ application, unlike the previously described two compounds, reference enzyme activities were similar to that of control in case of both passage numbers. In such conditions, ClO_2_ is supposed to have a gentle and transient effect, which could be easily tolerated by PDLSCs. The hyperpure ClO_2_ was found to be much less toxic than CHX or H_2_O_2_ and the PDLS cells were able to regenerate after hyperpure ClO_2_ treatment in opposite to CHX or H_2_O_2_. In accordance with this, the expression of stem cell markers was not significantly altered in the PDSC cultures.

## Conclusions

In conclusion, in the present study, impedimetry was successfully applied to register cell physiological activities of PDLSCs in a real-time mode. Our studies on cell viability demonstrated that ClO_2_ has no significant effect on the viability of PDLSCs in concentrations that are toxic for microbes and applied in dental clinical practice. That high selectivity of ClO_2_ killing the microbes while not hurting the much larger stem cells is due mostly to the size-selective property of ClO_2_. The killing time of a cell or a biofilm is proportional with the square of its characteristic dimension (Supplementary material).

Aging of PDLSC cultures had no significant effect on the responsiveness of cultures to antimicrobial agents tested either in cell survival or in the expression of the mesenchymal stem cell markers. The active ingredients of mouthwash (e.g., H_2_O_2_ and CHX) applied in the clinical routine for the endodontal or periodontitis management had a toxic effect on PDLSCs. On the contrary, the novel, hyperpure compound, ClO_2_ proved to be less toxic and the PDLSCs were able to regenerate after the treatment. Consequently, the hyperpure ClO_2_ seems to be an ideal compound and a superior substituent of the traditional antiseptic agents in the home and clinical dental care.

## Electronic supplementary material

ESM 1 (DOCX 37.8 kb)
