# Supplementary material for: Comparative study of hyperpure chlorine dioxide with two other irrigants regarding the viability of periodontal ligament stem cells
Source: Clin Oral Investig. 2020 Oct 12;25(5):2981–92. doi: 10.1007/s00784-020-03618-5 (PMC8060220; doi:10.1007/s00784-020-03618-5)
Supplement: Supplementary file 1 — (DOCX 37.8 kb) [file 784_2020_3618_MOESM1_ESM.docx]

**Supporting information**

**Explanation for the high selectivity of ClO_2_**

**between bacteria and PDL stem cells based on their different size**

The aim of this supplement is to show briefly how the numerical examples of the Conclusion (the killing time for an Escherichia coli bacterium or a PDL stem cell, both with a roughly cylindrical geometry, moreover for a planar layer of PDL stem cells) were calculated.

*Killing time for planar and cylindrical geometries*

The concept of killing time (T_KILL_) was introduced by Noszticzius et al. [1]. They have suggested that when chlorine dioxide penetrates into a protein medium, a very sharp reaction front appears due to the reaction-diffusion processes. This is because ClO_2_ reacts rapidly with three amino acids: cysteine [2], tyrosine [3] and tryptophan [4]. The reaction is also rapid with the residues of these amino acids in proteins. These are the major reactants or “substrates” of ClO_2_ in a cell because other organic components react much more slowly, or present in much smaller concentrations. When the ClO_2_ front penetrates into a spherical cell for example, the concentration of the unreacted substrates behind the inward moving sharp spherical front is practically zero while there is no ClO_2_ in the region ahead of the front. The rate of the reaction is controlled by the rate of the diffusion of ClO_2_ to the front, which is moving gradually toward the center of the sphere. Now, when the sharp front arrives to the center of the spherical cell, all reactive amino acids and amino acid residues have already reacted, thus the cell should be dead. Based on that picture the “killing time” is defined as either the time required for the front to reach the center of a spherical cell or - in case of other more general geometries - it is the time required by the ClO_2_ front to reach every parts of the cell and to consume all substrates there. Obviously, that is a rough approximation only (the cell may die much earlier) but applying the reaction-diffusion equations such times can be easily calculated for planar, cylindrical and spherical geometries [1] and can give a good order of magnitude estimate for the real killing times.

The result for a planar geometry is given by equation (3) of [1] (in the analogous equation (M) some indexes are renamed):

$T_{KILL,M}=\frac{1}{2}\cdot\frac{s}{c_{0}}\cdot\frac{\left( d_{M} \right)^{2}}{D}$ (M)

Here $T_{KILL,M}$ is the killing time for a membrane shaped planar cell with a thickness $d_{M}$, *s* is the substrate concentration in the membrane (the ClO_2_ consuming capacity of all the substrates in the unit volume of the medium ) and *D* is the diffusion coefficient of ClO_2_ within the medium of the membrane. The top of the planar cell is “open”: it is in contact with a well-stirred aqueous solution containing ClO_2_ in a fixed $c_{0}$ concentration. The bottom is “closed”, the planar membrane is in contact with a glass or a metal plate (ClO_2_ is not able penetrate into these media). At the initial moment (time zero), there is no ClO_2_ within the membrane, thus the planar reaction front starts at the top and moves toward the bottom, which is reached at the time $T_{KILL,M}$ when all substrates of the cell are consumed.

The result for a cylindrical geometry is given by equation (4) of [1] (in the analogous equation (C) below, some indexes are renamed again):

$T_{KILL,C}=\frac{1}{16}\cdot\frac{s}{c_{0}}\cdot\frac{\left( d_{C} \right)^{2}}{D}$ (C)

In eq. (C) $T_{KILL,C}$ is the killing time for a cylindrical cell with a diameter $d_{C}$, *s* is the substrate concentration in the cylinder and *D* is the diffusion coefficient of ClO_2_ within the medium of the membrane. The cylindrical surface of the cell is “open”: it is in contact with a well-stirred aqueous solution containing ClO_2_ in a fixed $c_{0}$ concentration. At the initial moment (time zero), there is no ClO_2_ within the cylindrical cell, thus the cylindrical reaction front starts at the surface and moves inward to the axis of the cylinder, which is reached at the time $T_{KILL,C}$ when all substrates of the cell are consumed.

*Estimating “s” and “D”, the two non-geometric cellular parameters*

Cells can differ not only in their size and in geometry, but also in their chemical composition. In our equations, the effect of chemical composition is taken into account by the substrate concentration *“s”*. Noszticzius et al. [1] have determined that parameter in two different protein membranes at laboratory temperature (22-24 °C). The first one was an artificial membrane (gelatine) and the second was a natural one (pig bladder). Their results: *s(gelatine) =* 26.2 mM, *s(pig bladder) =* 56 mM.

Diffusion coefficients also depend on the chemical composition of the medium. In the present case the dependence of *“D”* on the chemical composition of the medium is rather weak, however. It was found [S1] that the diffusion coefficient of ClO_2_ in a cellular pig bladder tissue

(*D(pig bladder)* = 1.8×10^–6^ cm^2^s^–1^ ) is only 30 % smaller than in the unstructured gelatine gel medium (*D(gelatine) =* 2.4×10^–6^ cm^2^s^–1^ ). This is because ClO_2_ is soluble both in aqueous and in lipid media thus lipid membranes are not able to modify substantially the diffusional transport of ClO_2_. Consequently, to estimate the killing times at room temperature, we will use the same diffusion coefficient in the formula (C) and (M), which was measured in a pig bladder membrane. This way we will have order of magnitude estimates only but that is still enough to show the several orders of magnitude difference in the killing time of a bacteria and a human cell.

*Calculating the killing time for a single cylindrical bacterium (Escherichia coli).*

*Comparison of calculated and measured values.*

The killing time of a 1 μm diameter cylindrical bacterium was calculated by Noszticzius et al. [1] for a ClO_2_ concentration *c_0_* = 300 mg/L (4.45 mM) using formula (C) and applying the pig bladder parameters. Their result is 4.4 ms. As the geometry and the numerical value of the diameter fits to Esherichia coli (a bacterium with cylindrical symmetry with 1µm diameter [5,]), that estimate is valid for an E.coli bacterium as well. In the present experiments the ClO_2_ concentrations (25 and 2.5 mg/L) are smaller, however, thus the killing times predicted by equation (C) should be proportionally (12 and 120 times) larger

$$T_{KILL,E.coli}\left( c_{0}=25 {mg}/L \right)=53 ms$$

$$T_{KILL,E.coli}\left( c_{0}=2.5 {mg}/L \right)=530 ms$$

These times are 10^3^ or 10^4^ shorter than the 10 minutes time of the ClO_2_ treatment applied on PDLSCs. Consequently, there is no chance for an E. coli bacterium to survive a ClO_2_ treatment, which was, on the other hand, survived by the PDL stem cells according to our experiments.

Before calculating the theoretical killing times for a PDLSC, however, it is reasonable to check the validity of these calculations for E. coli by comparing calculated and measured killing times for that bacterium. As we could see the calculated killing times for E. coli are very short for 25 and 2,5 mg/L ClO_2_ concentrations, thus it would be technically difficult to perform such fast measurements. We could find, however, some experimental data in the literature for the kinetics of the killing in 0.25 mg/L ClO_2_  at 20 °C [6]. Our calculations for 0.25 mg/L ClO_2_  but for a somewhat warmer temperature (22-24 °C ) predict a 5.3 s killing time for that concentration. Benarde et al. [6] write about their experimental observations: “To obtain 99% kill, a concentration of 0.25 mg/L required …41 sec at 20 °C and 16 sec at 30 °C…” These data show that the killing of E. coli in a 0.25 mg/L ClO2 solution is really fast, but to estimate an experimental killing time based on their data is not easy. On the basis of the killing time concept one would expect a nearly constant or slowly decreasing population of the bacteria which drops sharply around the killing time. In contrast Fig. 6. of Benarde et al. [6] show long tailed curves suggesting a wider spectrum of killing times. This is hard to understand in the case of a population consisting of single planktonic bacteria exclusively but can be explained if, beside the single bacteria, we assume various bacterium clusters containing 2, 3, 4, or more bacteria. Such clusters can appear in growing E. coli colonies [7]. In that case the presence of the clusters can give an explanation for the observed spectrum of killing times. Larger clusters require larger killing times. If the appearance of the sharp drop in the curve is regarded as an “experimental killing time” for the “monomeric” bacterium, then this value at 20 °C is about 15± 5 seconds that is about 3 times larger than our theoretical estimate. The difference is probably due to low molecular mass substrates (e.g. cysteine and thiols like glutathione [8]) which are present and also produced continuously in a living E. coli but which are missing from the dead cells of the pig bladder (where both the small molecules and also the mechanism producing them are missing).

*Calculating the killing time for a single PDLS cell*

We have already theoretical killing time estimates for a cylindrical bacterium (E.coli) with a diameter of 1 μm. Regarding Eq. (C), and knowing that the PDLS cells have also a cylindrical geometry, but with a diameter of 50 μm, we are able to calculate the killing time estimates for those cells as well, if we assume that the two non-geometric parameters (*s* and *D*) are the same (or at least they are not very much different) in a pig bladder membrane, in an E. coli, or in a PDLSC. As the size is 50 times larger, the killing times for a PDLSC should be 2500 times larger:

$$T_{KILL, PDLSC}\left( c_{0}=25 {mg}/L \right)\approx130 s \approx2 min$$

$$T_{KILL, PDLSC}\left( c_{0}=2.5 {mg}/L \right)\approx1320 s \approx22 min$$

The above theoretical estimate shows that a single PDLS cannot survive a 10 minute treatment in a 25 mg/L ClO_2_ bath at 22-24 °C . However, the stem cells in our experiments are present not as single planktonic cells, but as a layer of cells (see e.g. Fig. 3 A). Thus it is reasonable to calculate the killing time for that geometry also.

*Calculating the killing time for a 25 micron thick layer of PDLSCs*

PDLSCs form roughly a monolayer on the surface of the measuring electrodes. When the cells are attached to the electrodes their originally circular cross section is somewhat deformed and becomes oval. As a consequence the monolayer thickness is only about half of the original 50 μm diameter. Thus we have to calculate with a 25 μm thick membrane of stem cells. Dividing equation (M) with equation (C) and taking into account that $d_{M}={d_{C}}/2$ we obtain:

$$T_{KILL,M}=2\cdot T_{KILL,C}$$

Thus

$$T_{KILL, PDLSC-LAYER}\left( c_{0}=25 {mg}/L \right)\approx260 s \approx4 min$$

$$T_{KILL, PDLSC-LAYER}\left( c_{0}=2.5 {mg}/L \right)\approx2640 s \approx44 min$$

*Comparing the results of the PDLSC viability measurements with the theoretical estimates*

As Fig. 1 shows, a part of the PDLSCs can survive the 10 minute treatment in a 25 mg/l ClO_2_ solution even at 37 °C. In contrast, the theoretical killing time calculated for a PDLSC layer in a 25 mg/l ClO_2_ solution is only 4 min at 24 °C, and most probably, it would be even shorter at 37 °C. (Unfortunately, the exact time cannot be calculated, as we have no reliable non-geometric parameter values for that higher temperature.) It is true, that two days after the treatment in a 25 mg/l solution the normalized Cell Index (CI) is significantly lower (about 0.5 see Fig. 1 B) compared to the case when the treatment was made in a 2.5 mg/l solution (CI ≈ 0.8), but it is still not zero. Thus the theoretical killing time is too short, the cells can survive longer than expected.

As we could see the killing time for a living E. coli bacterium was also underestimated by a factor of 3. Applying that number as a correction factor would give a 12 min killing time at 24 °C but, most probably, that time would be significantly shorter at 37 °C. It is clear that while the simple formula (C) predicts correctly the order of magnitude differences between the killing times of small and large living cells, the simple model developed for the penetration of a sharp reaction front into a non-living protein medium underestimates the numerical values for living cells by a factor of 3, or even more. From the point of the ClO2 penetration the main difference between a non living animal tissue and living cells is the presence and continuous production of small thiol containing molecules [8], like glutathione for example in the living cells, which can react with, and consume ClO_2_ rapidly [2]. Such small molecules are absent from and are not produced in dead tissues. Regarding the special case of stem cells it is interesting to remark in this respect, that to maintain stem cell function these cells require high glutathione levels [9].

**References**

1. Noszticzius Z, Wittmann M, Kály-Kullai K, Beregvári Z, Kiss I, Rosivall L, Szegedi J. Chlorine dioxide is a size-selective antimicrobial agent. PLoS ONE. 2013;8(11): e79157. <https://doi.org/10.1371/journal.pone.0079157>

2. Ison A, Odeh IN, Margerum DW. Kinetics and mechanisms of chlorine dioxide and chlorite oxidations of cysteine and glutathione. Inorganic Chemistry. 2006; 45:8768–75. <https://doi.org/10.1021/ic0609554>

3. Napolitano M J, Green BJ, Nicoson JS, Margerum DW. Chlorine dioxide oxidations of tyrosine, N-acetyltyrosine, and Dopa. Chemical Research in Toxicology. 2005; 18:501–8. <https://doi.org/10.1021/tx049697i>

4. Stewart DJ, Napolitano MJ, Bakhmutova-Albert EV, Margerum DW. Kinetics and mechanisms of chlorine dioxide oxidation of tryptophan. Inorganic Chemistry. 2008; 47: 1639–47. <https://doi.org/10.1021/ic701761p>

|  |  |
| --- | --- |

5. Reshes G,Vanounou S,Fishov I, Feingold M. Cell shape dynamics in Escherichia coli. Biophysical Journal. 2008; 94: 251–64. doi:[10.1529/biophysj.107.104398](https://dx.doi.org/10.1529%2Fbiophysj.107.104398)

6. Benarde MA, Snow WB, Oliveri VP, Davidson B. Kinetics and mechanism of bacterial disinfection by chlorine dioxide. Applied Microbiology. 1967; 15(2):257-65.

PMID: 5339839

7. <https://en.wikipedia.org/wiki/Escherichia_coli#/media/File:E_coli_at_10000x,_original.jpg>

8. Poole LB. The basics of thiols and cysteines in redox biology and chemistry. Free Radical Biology and Medicine. 2015; 0:148–57. <https://doi.org/10.1016/j.freeradbiomed.2014.11.013>

9. Jeong EM, Yoon JH, Lim J, Shin JW, Cho AY, Heo J et al.

Real-time monitoring of glutathione in living cells reveals that high glutathione levels are required to maintain stem cell function. Stem Cell Reports. 2018; 10:600–14. doi: [10.1016/j.stemcr.2017.12.007](https://dx.doi.org/10.1016%2Fj.stemcr.2017.12.007)
